# Is adherence to the 24-hour movement guidelines related to a lower risk of cardiovascular events? A prospective cohort study including 13 897 participants from the SUN project

**DOI:** 10.1093/eurpub/ckag191

**Published:** 2026-10-24

**Authors:** Alexander Finnemore, Reyes Viñals Soler, Maira Bes‐Rastrollo, Miguel Ángel Martínez-González, Alejandro Fernandez-Montero

**Affiliations:** Department of Occupational Medicine, University of Navarra, Pamplona, Spain; Department of Occupational Medicine, University of Navarra, Pamplona, Spain; Department of Preventive Medicine and Public Health, School of Medicine, University of Navarra, Pamplona, Spain; CIBER Fisiopatología de la Obesidad y Nutrición (CIBER Obn), Instituto de Salud Carlos III, Madrid, Spain; Navarra Institute for Health Research (IdiSNA), Pamplona, Spain; Department of Preventive Medicine and Public Health, School of Medicine, University of Navarra, Pamplona, Spain; CIBER Fisiopatología de la Obesidad y Nutrición (CIBER Obn), Instituto de Salud Carlos III, Madrid, Spain; Navarra Institute for Health Research (IdiSNA), Pamplona, Spain; Department of Nutrition, T.H. Chan School of Public Health, Harvard University, Boston, United States; Department of Occupational Medicine, University of Navarra, Pamplona, Spain; Department of Nutrition, T.H. Chan School of Public Health, Harvard University, Boston, United States

## Abstract

Cardiovascular disease (CVD) remains the leading global cause of death, and preventive interventions are likely to be the most effective strategies. The 24-hour movement guidelines emphasize an integrated score, including physical activity (PA), sedentary time, and sleep. The combined effect of adhering to all three 24-hour movement guidelines on CVD events remains unclear. In a prospective assessment of 13 897 Spanish university graduates from the Seguimiento Universidad de Navarra cohort, the relationship between a comprehensive exposure (adherence to all three 24-hour movement guidelines) and CVD incidence was investigated. Participants completed baseline questionnaires from 1999, with biennial follow-up. Exposure was assessed using the Canadian 24-Hour Movement Guidelines: ≥ 150 minutes/week of moderate-to-vigorous PA, ≤ 8 hours/day sitting, and 7–9 hours/night sleep (or 7–8 hours for ≥ 65 years). A composite score (0–3 points) was calculated for the exposure. The primary endpoint was incident CVD (nonfatal myocardial infarction, stroke, cardiovascular death). Nearly half (46.68%) of participants met all three guidelines, while only 0.96% met none. Over a mean follow-up of 14.48 years, 148 cardiovascular event (CVE) occurred. Fully adjusted models revealed a significantly reduced CVE risk for participants meeting two [hazard ratio (HR) = 0.37; 95% confidence interval (CI): 0.14–0.99], or three (HR = 0.36; 95% CI: 0.14–0.95) guidelines compared to meeting none. Adherence to at least two of the 24-hour movement guidelines appears to be associated with a lower risk of CVEs. These findings suggest that a comprehensive approach to daily movement behaviors may play a beneficial role in CVD prevention. Public health strategies could consider promoting these integrated guidelines as part of broader efforts to improve cardiovascular health.

## Introduction

Cardiovascular disease (CVD) is the leading cause of death worldwide [1, 2]. Despite advances in treatment, prevention remains the most effective strategy [3]. Physical activity (PA) is widely recognized as a key factor in cardiovascular health, associated with lower CVD risk, improved metabolic function, and reduced mortality [4, 5].

The 24-hour movement framework proposes a broader perspective, integrating three essential behaviors: PA, sedentary time, and sleep [6]. Global health organizations recommend at least 150–300 minutes of moderate-intensity or 75–150 minutes of vigorous-intensity exercise per week. Yet, movement alone does not tell the whole story [7]. Long periods of inactivity increase metabolic dysfunction [8]. Poor sleep disrupts cardiovascular regulation [9]. Taken together, these behaviors reflect a simple yet fundamental idea: how people spend their entire day matters. They do not act in isolation; they form a cycle, reinforcing or impairing health in ways that single-focus recommendations often miss [6].

The Canadian 24-Hour Movement Guidelines for Adults, released in 2020, were the first adult guidelines to bring the three behaviors together into a single set of recommendations covering the whole day. For adults aged 18–64 years, they recommend at least 150 minutes per week of moderate-to-vigorous aerobic activity, muscle-strengthening activities involving major muscle groups at least twice a week, several hours of light activity including standing, no more than 8 hours per day of sedentary time (of which no more than 3 hours of recreational screen time) with frequent interruption of prolonged sitting, and 7–9 hours of good-quality sleep with consistent bed and wake times. For adults aged 65 years or older, the recommended sleep duration is 7–8 hours and activities that challenge balance are added. What distinguishes this framework from behavior-specific recommendations is its underlying premise that the day is a finite whole: time is reallocated rather than created, so an increase in one behavior necessarily comes at the expense of another. The greatest benefit is therefore expected when sedentary time is replaced by PA and light activity by moderate-to-vigorous activity, while adequate sleep is preserved [10, 11].

Research suggests that meeting all three guidelines—sufficient PA, limited sedentary time, and adequate sleep—offers stronger protection than any one behavior alone [12]. The Canadian 24-Hour Movement Guidelines have linked full adherence to lower all-cause mortality [12], but its specific impact on CVD events remains uncertain [12]. Given that cardiovascular conditions account for a significant share of global deaths, understanding whether comprehensive movement adherence improves survival is essential. Moreover, whether a dose–response effect exists, where greater adherence yields progressively lower risk, remains unexplored.

This study examines the relationship between adherence to the 24-hour movement guidelines and CV events in a prospective cohort. By assessing different levels of compliance, it aims to determine whether meeting all three movement guidelines provides stronger protection than focusing on PA alone. The findings will contribute to a shift in how preventive strategies are framed, moving from isolated recommendations to a full-day approach that recognizes the interdependence of movement, rest, and health.

## Methods

### Study design and population

This study was conducted within the Seguimiento Universidad de Navarra (SUN) cohort, a prospective, ongoing cohort designed to assess the role of lifestyle and dietary habits in chronic disease development among Spanish university graduates. The study began in 1999, with biennial follow-up questionnaires collecting information on participants’ health, lifestyle, and medical diagnoses. Further details on the cohort profile are available at www.proyectosun.es. The SUN Project was approved by the Institutional Review Board of the University of Navarra (091/2008). Voluntary completion of the baseline questionnaire was considered to imply informed consent, and participants were informed of their right to refuse participation or to withdraw their consent at any time, in accordance with the principles of the Declaration of Helsinki.

Participants who completed the baseline questionnaire (Q_0_) from 1 December 1999 to 31 March 2024 were included. Subsequently, those with insufficient follow-up time (*n* = 186), implausible energy intake values [13] (*n* = 2141), or missing data on key exposure variables (*n* = 5776) were excluded. Additionally, 205 participants with pre-existing CVD at baseline were removed, leaving a total of 15 013 participants. Of these, 1116 were lost to follow-up, resulting in a final sample of 13 897 participants and a retention rate of 92.57% (Figure S1).

### Exposure assessment: 24-hour movement guidelines

The Canadian 24-Hour Movement Guidelines for Adults offer integrated recommendations for PA, sedentary behavior, and sleep, tailored for two age groups: 18–64 years and 65 years or older [10]. These guidelines underscore that all movement behaviors throughout a 24-hour period are interconnected and collectively contribute to overall health. For both age groups, a healthy day encompasses at least 150 minutes per week of moderate-to-vigorous aerobic PA, along with muscle-strengthening activities for major muscle groups performed at least twice a week. Several hours of light physical activities, including standing, are also recommended. Regarding sedentary behavior, the guidelines advise limiting total sedentary time to 8 hours or less, which includes no more than 3 hours of recreational screen time, and emphasize breaking up long periods of sitting as often as possible. For sleep, adults aged 18–64 years should aim for 7–9 hours of good-quality sleep on a regular basis with consistent bed and wake-up times, while those aged 65 years or older should target 7–8 hours of good-quality sleep with consistent bed and wake-up times. Additionally, adults aged 65 years or older are specifically advised to include physical activities that challenge balance. A central tenet of these guidelines is that replacing sedentary behavior with additional PA and trading light PA for more moderate-to-vigorous PA, while maintaining sufficient sleep, can lead to greater health benefits [10, 11].

PA was assessed using a validated 17-item self-administered questionnaire [14], which collected information on common leisure-time activities such as walking, jogging, cycling, swimming, and team sports. Participants reported both the frequency and duration of these activities, accounting for seasonal variations. Each activity was assigned a metabolic equivalent of task (MET) value based on the Compendium of Physical Activity [15]. Weekly hours per activity were calculated and converted into daily averages. PA intensity was classified as either light-to-moderate (3–<6 METs) or vigorous (≥ 6 METs). Adherence to PA guidelines was defined as engaging in at least 150 minutes per week of moderate-to-vigorous PA or 75 minutes per week of vigorous PA, or a combination of both. These guidelines are the same as those recommended by the WHO [7]. To further refine our assessment, walking speed was self-reported using four predefined categories—slow, moderate, brisk, and very brisk [16]—and corresponding MET values were assigned to improve the estimation of total PA.

Sedentary behavior was evaluated using self-reported daily sitting time, which included hours spent watching television, using a computer, or sitting while driving. To obtain an overall measure of sedentary time, participants reported their television viewing separately for weekdays and weekends, and a weighted mean was calculated as (5× weekday hours + 2× weekend hours)/7. Adherence to sedentary behavior guidelines was defined as spending no more than 8 hours per day sitting.

Sleep duration was assessed through two questions: “How many hours per night do you sleep on weekdays?” and “How many hours per night do you sleep on weekends?” A weekly average was derived using the same weighted formula applied to sedentary behavior. Validation of self-reported data has been conducted in a subsample of the cohort [17]. Sleep adherence was classified according to age-based recommendations, with optimal sleep duration considered to be seven to nine hours per night for adults younger than 65 years and 7–8 hours per night for those aged 65 or older.

A composite 24-hour movement score was calculated by summing adherence across the three behavioral components, with one point assigned for meeting PA guidelines, one point for staying within the recommended sedentary behavior limits, and one point for achieving age-appropriate sleep duration. The resulting score ranged from 0 to 3, with higher values indicating greater alignment with 24-hour movement recommendations.

### Outcome assessment: cardiovascular events

The primary endpoint for this study was the incidence of CVD events, including nonfatal myocardial infarction (MI) and stroke events, as well as cardiovascular death including stroke and MI. Information on these events was obtained through active follow-up and record linkage with the Spanish National Death Index. Participants who failed to respond to five consecutive follow-ups were contacted via phone or email. Event status was verified through reports from family, work associates, professional associations, and postal authorities. Causes of events and deaths were confirmed using medical reports and death certificates, cross-checked with National Statistical Institute data. Cardiovascular events (CVEs) were classified according to ICD-10 codes by a review of medical records by expert cardiologists. The most recent event confirmation was conducted in December 2022.

### Covariates and confounders

Baseline characteristics included age, sex, body mass index (BMI), smoking status, alcohol intake, total energy intake, adherence to the Mediterranean diet, education level, and comorbidities (insomnia, sleep apnea, hypertension, diabetes, cancer, and previous CVE including MI, angina, coronary bypass, stroke). Dietary intake was assessed with a semi-quantitative 136-item food-frequency questionnaire previously validated in Spain and in this cohort [18]. Adherence to the Mediterranean diet was summarized with the 0–9-point Mediterranean Diet Score proposed by Trichopoulou *et al*. [19], which assigns one point each for intakes above the sex-specific median of vegetables, fruits and nuts, legumes, cereals, fish, and the ratio of monounsaturated to saturated fatty acids; one point each for intakes below the sex-specific median of meat and dairy products; and one point for moderate alcohol consumption, with higher scores indicating closer adherence.

### Statistical analysis

Time-to-event analyses were conducted using Cox proportional hazards models, estimating hazard ratios (HRs) with 95% confidence intervals (CIs). The reference group consisted of participants not meeting any of the 24-hour movement guidelines.

Statistical analyses were performed using STATA, with statistical significance set at *P* < .05. A Cox regression was then conducted, and results are expressed as HRs and 95% CIs. Results were calculated crude, adjusted for age and sex, and multivariable-adjusted. Multivariable-adjusted analyses were also conducted for each type of CVD event (MI, stroke, or CVD death) and for each component of the 24-hour score, considering participants who did not meet the respective threshold as the reference group and additionally adjusting for the other components of the 24-hour score. Adjustments in all analyses included age, sex, BMI, lifetime smoking, alcohol intake, education, comorbidities, and total energy intake. Stratified analyses further explored potential effect modification by age and sex.

## Results

At baseline, the mean age of participants was 37.12 years (SD = 11.45), and 61.47% were female. Table 1 presents the distribution of participants according to their adherence to the 24-hour movement guidelines. The proportion of individuals meeting none, one, two, and all three movement guidelines was 0.96%, 11.86%, 40.50%, and 46.68%, respectively. Notable differences across these adherence groups include the proportion of women, which increases with higher guideline adherence, from 47.76% in the “None” group to 62.42% in the “Two” group and 61.37% in the “Three” group. Conversely, mean BMI generally decreases as adherence rises, with the “None” group averaging 25.0 kg/m^2^ compared to 23.2 kg/m^2^ in the “Three” group. Similarly, participants meeting more guidelines tend to have fewer pack-years of smoking, decreasing from 10.0 pack-years in the “None” group to 5.2 in the “Three” group. Furthermore, the prevalence of both insomnia and sleep apnea shows a considerable decrease with increased guideline adherence; insomnia dropped from 30.6% in the “None” group to 15.6% in the “Three” group, and sleep apnea fell from 3.73% to 1.55% across the same categories. Over a mean follow-up period of 14.48 years (SD = 5.98), a total of 148 incident cases of CVE were documented.

**Table 1:** Baseline characteristics of participants according to number of 24-hour movement guideline factors (The SUN Project, 1997–2024).^a^

Baseline characteristics	24-hour movement guidelines met			
	None	1	2	3
Participants, *n*	134	1648	5628	6487
Sex, women (%)	47.76	59.77	62.42	61.37
Age (years)	37.4 (11.9)	36.7 (11.2)	36.9 (11.4)	37.5 (11.5)
Educational level (years)	5.2 (1.5)	5.2 (1.5)	5.1 (1.5)	5.1 (1.5)
Body mass index (kg/m^2^)	25.0 (4.1)	24.0 (3.9)	23.5 (3.6)	23.2 (3.3)
Mediterranean diet pattern	3.8 (1.7)	4.0 (1.7)	4.1 (1.8)	4.3 (1.8)
Alcohol consumption (g/d)	10.4 (16.8)	6.8 (11.5)	6.6 (10.2)	6.5 (9.5)
Smoking (pack-years)	10.0 (13.9)	5.9 (9.7)	5.7 (9.5)	5.2 (8.8)
Diet (%)	9.70	7.71	7.91	7.54
Caloric intake	2333 (676)	2301 (605)	2346 (612)	2380 (602)
Snoring (%)	21.64	21.91	18.55	14.83
Insomnia (%)	30.60	21.0	19.88	15.59
Sleep apnea (%)	3.73	2.43	1.74	1.55
Prevalent diabetes (%)	1.49	1.58	1.63	1.48
Prevalent hypertension (%)	11.94	10.38	9.79	8.83
Prevalent cancer (%)	4.48	2.37	2.35	2.42

a Values are expressed as mean (SD) unless otherwise noted.

Table 2 summarizes the risk of CVE in relation to the number of 24-hour movement factors adhered to, with HRs provided for crude, age- and sex-adjusted, and fully adjusted. In the fully adjusted model, those meeting one movement guideline had a 60% lower risk of CVE (HR = 0.40; 95% CI: 0.14–1.13), two guidelines were associated with a 63% lower risk of CVE (HR = 0.37; 95% CI: 0.14–0.99), and adherence to all three guidelines resulted in a 64% reduced risk of CVE (HR = 0.36; 95% CI: 0.14–0.95) when compared to participants who did not meet any of the guidelines.

**Table 2:** Risk of CVE, according to number of 24-hour movement guideline factors (The SUN Project).

	24-hour score				For each additional factor
	0	1	2	3	
Person-year	1923	23 883	81 516	93 901	
Incident cases	5	18	62	63	
Crude	1 (ref.)	0.32 (0.12–0.85)	0.29 (0.11–0.71)	0.25 (0.10–0.62)	0.80 (0.65–0.99)
Adjusted for age and sex	1 (ref.)	0.36 (0.13–0.98)	0.34 (0.13–0.85)	0.29 (0.12–0.73)	0.82 (0.66–1.02)
Multivariable adjustment^a^	1 (ref.)	0.40 (0.14–1.13)	0.37 (0.14–0.99)	0.36 (0.14–0.95)	0.88 (0.70–1.10)

a Adjusted for age, sex, educational level, BMI, smoking, calorie intake, alcohol intake, following a special diet, adherence to Mediterranean diet, snoring, insomnia, sleep apnea, prevalence of cancer, prevalence of hypertension, prevalence of diabetes mellitus.

Table 3 presents the association between adherence to the movement guidelines and specific CVE, including MI, stroke, and overall CVD mortality. The analysis was adjusted for the same covariates as in Table 2. For CVD mortality, participants adhering to at least one guideline exhibited a substantially lower risk compared to those with no adherence (HR = 0.02; 95% CI: 0.00–0.42). A similar pattern was observed for MI and stroke events, where adherence to any guideline was associated with lower risk estimates, but with wider CIs.

**Table 3:** Risk of different causes of CVE and CVD mortality, according to the number of 24-hour movement guideline factors (The SUN Project).

	24-hour score			
	0	1	2	3
Person-year	1923	23 883	81 516	93 901
MI events	2	7	22	32
Multivariable-adjusted HR^a^ (95% CI)	1 (ref.)	0.43 (0.08–2.33)	0.40 (0.08-1.95)	0.60 (0.13–2.95)
Stroke events	1	10	20	20
Multivariable-adjusted HR^a^ (95% CI)	1 (ref.)	1.55 (0.17–14.43)	0.80 (0.09–7.18)	0.72 (0.08–6.43)
CVD mortality	2	1	20	11
Multivariable- adjusted HR^a^ (95% CI)	1 (ref.)	0.02 (0.00–0.42)	0.23 (0.04–1.30)	0.14 (0.02–0.80)

a Adjusted for age, sex, educational level, BMI, smoking, calorie intake, alcohol intake, following a special diet, adherence to the Mediterranean diet, prevalence of cancer, sleep apnea, snoring, insomnia, prevalence of hypertension, prevalence of diabetes mellitus.

Figure 1 presents the associations between adherence to each individual component of the 24-hour movement guidelines and CVE risk. Meeting the physical activity recommendation was associated with a significantly lower risk of CVE (HR = 0.70; 95% CI: 0.55–0.89), whereas adherence to the sedentary time (HR = 0.95; 95% CI: 0.77–1.18) and sleep recommendations (HR = 0.89; 95% CI: 0.71–1.13) showed no statistically significant associations.

## Discussion

This prospective cohort study of 13 897 participants from the SUN Project over a mean follow-up of 14.48 years demonstrates a significant protective effect of meeting comprehensive 24-hour movement guidelines on CVE incidence. The critical finding is that adherence to at least two of the three guidelines (PA, sedentary time, and sleep) is associated with substantial risk reduction, with the most robust protection observed when meeting two or three guidelines simultaneously. This suggests that the threshold for significant cardiovascular protection lies in adopting a multifaceted approach to daily movement behaviors rather than focusing on individual components in isolation.

Our results align with existing research advocating for a holistic perspective on movement behaviors. The Canadian 24-Hour Movement Guidelines have similarly demonstrated that comprehensive adherence is linked to lower all-cause mortality [20]. However, our study extends this evidence specifically to CVEs, providing crucial support for the integrated framework approach. The strength of our findings lies in the comprehensive assessment of all three core movement components rather than examining them separately, recognizing that these behaviors form an interconnected cycle that collectively influences physiological processes and health outcomes [6].

The observed associations are biologically plausible and consistent with established CVD mechanisms. PA improves cardiovascular fitness, reduces inflammation, and enhances metabolic health [3, 21]. Adequate sleep supports cardiovascular recovery and hormonal regulation, while reduced sedentary time prevents the metabolic dysfunction associated with prolonged sitting [9]. The dose–response relationship observed, in which greater adherence yields progressively lower risk, supports a causal interpretation. Furthermore, the consistency of protective effects across different levels of adherence strengthens the evidence for causality, as does the coherence with existing epidemiological knowledge about individual lifestyle factors and cardiovascular risk [22].

Several strengths enhance the validity of our findings. The prospective cohort design minimizes recall bias and establishes temporal relationships. The comprehensive adjustment for multiple confounding factors, including demographic, lifestyle, and clinical variables, reduces the likelihood that observed associations are due to other health-related behaviors or pre-existing conditions. The long follow-up period and use of validated questionnaires further strengthen the evidence quality.

Despite these strengths, several limitations warrant consideration. The self-reported nature of movement behaviors could introduce measurement error, though validated instruments were used to minimize this concern. The relatively low number of incident CVEs, particularly for specific endpoints such as MI and stroke, limited statistical power for these analyses, as evidenced by the wider CIs for specific outcomes.

The study population of Spanish university graduates may not be fully representative of the general population, potentially limiting generalizability to other demographic groups. Additionally, despite extensive adjustment, residual confounding by unmeasured variables (such as psychosocial or genetic factors) remains possible. Selection bias may also be present, given that participants were volunteers with potentially more favorable health profiles than the general population. The small number of events is itself informative about the nature of the cohort. All SUN participants are university graduates, and higher educational attainment is consistently associated with healthier behaviors, better access to preventive care, and lower cardiovascular morbidity and mortality, so a low event count is the expected consequence of studying this population rather than an incidental feature of our sample. We recorded 148 CVEs over 201 223 person-years, an incidence of approximately 74 events per 100 000 person-years, with 31 per 100 000 person-years for MI and 25 per 100 000 person-years for stroke. Population-based Spanish registries covering broadly comparable ages report substantially higher figures: in the REGICOR registry the incidence of MI among people aged 35–64 years was 169 per 100 000 per year in men and 28 per 100 000 per year in women [23], and the IBERICTUS study reported an age-standardized stroke incidence of 147 per 100 000 per year [24]. Even allowing for the young mean age of our participants at baseline and for the exclusion of prevalent CVD, our rates are roughly two to three times lower than those expected in the general Spanish population over the same period. This is consistent with a healthy-volunteer effect and with the socioeconomic and educational profile of the cohort, and it accounts for both the limited statistical power for individual endpoints and the width of our CIs. It also means that our estimates of absolute risk should not be extrapolated to less advantaged populations, in whom the absolute benefit of meeting the guidelines would plausibly be larger.

These findings have important implications for public health policy and clinical practice. The results support promoting comprehensive 24-hour movement guidelines rather than focusing solely on individual components like PA. Public health initiatives should emphasize the interconnected nature of movement behaviors and encourage simultaneous attention to PA, sedentary time, and sleep quality. Clinical recommendations should consider adopting these integrated guidelines to maximize population health impact.

Future research should incorporate objective measures of movement behaviors (such as accelerometry) and larger, more diverse cohorts to confirm these associations across different populations. Meta-analytic approaches may be necessary to achieve sufficient statistical power for specific cardiovascular endpoints. Studies examining the optimal combinations of guideline adherence and potential threshold effects would further inform evidence-based recommendations.

## Conclusion

Adherence to at least two of the 24-hour movement guidelines is significantly associated with reduced CVE risk in this prospective cohort. These findings highlight the importance of adopting an integrated approach to daily movement behaviors for CVD prevention. The evidence supports comprehensive public health strategies that address PA, sedentary time, and sleep as interconnected pillars of cardiovascular health.

**Figure 1:**
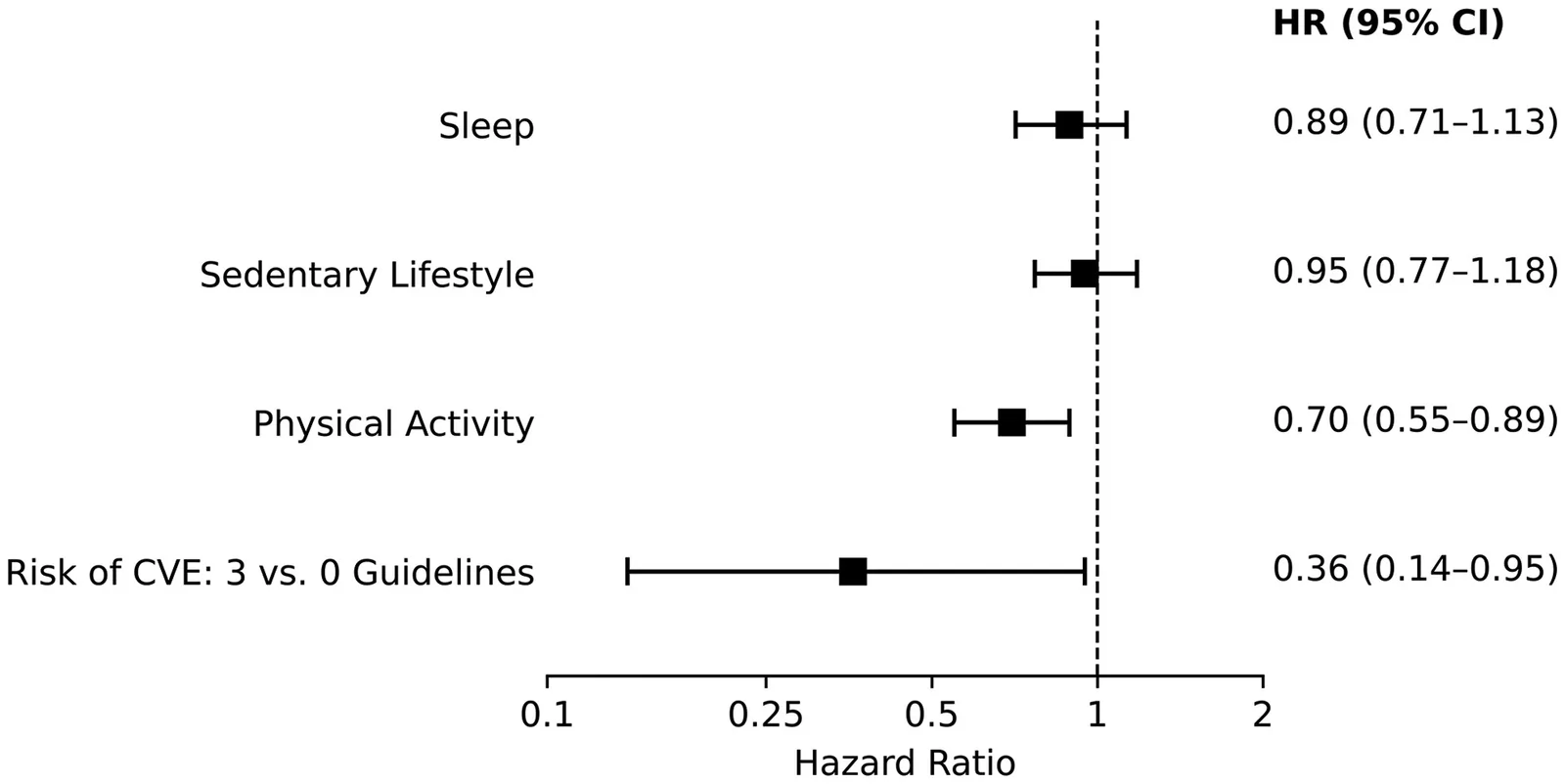
Multiadjusted HRs for the association between specific 24-hour movement behavior components (sleep, sedentary lifestyle, physical activity) and CVE. For the “sleep” group, the HR is 0.89 (95% CI: 0.71–1.13). The “sedentary lifestyle” group shows an HR of 0.95 (95% CI: 0.77–1.18). The “physical activity” group presents an HR of 0.70 (95% CI: 0.55–0.89). For meeting all three guidelines compared with none, the HR is 0.36 (95% CI: 0.14–0.95). HRs are multivariable adjusted.

## Supplementary Material

ckag191_Supplementary_Data

## Data Availability

The data underlying this article cannot be shared publicly owing to the privacy of the participants and applicable data protection regulations. The data will be shared on reasonable request to the corresponding author, subject to approval by the SUN Project investigators. CVD remains the leading global cause of death, and prevention is the most effective strategy. The 24-hour movement guidelines combine physical activity, sedentary time, and sleep into an integrated score. In the SUN cohort (13 897 participants, mean follow-up 14.5 years), adherence to these guidelines was assessed against cardiovascular event (CVE) incidence. Meeting at least two guidelines significantly reduced CVE risk (HR 0.37 and 0.36 vs. none), with nearly half the cohort adhering to all three. Public health implication: promoting a comprehensive approach to daily movement behaviors (PA, reduced sedentary time, and adequate sleep) may substantially lower CVD risk.
